# Portrayals of Medical Research in Prime-Time Dramas: A Content Analysis

**DOI:** 10.7759/cureus.112794

**Published:** 2026-07-16

**Authors:** Nicholas J Mulder, Chelsea C Lopez, Cecilia C Li, Jenny K Haely, Olivia C Manke, Mariela C Martinez, Michelle A Padley, Jeffrey S Jones

**Affiliations:** 1 Emergency Medicine, Michigan State University College of Human Medicine, Grand Rapids, USA; 2 Emergency Medicine, Corewell Health Medical Group, Grand Rapids, USA

**Keywords:** content analysis, medical dramas, medical research portrayal, prime-time television, public perception of science

## Abstract

Background: Television (TV) dramas are a significant source of information about health and medical research for lay audiences, blending accurate depictions with dramatic distortions that can shape beliefs about how research is conducted and governed. This study systematically analyzed selected U.S. television dramas to characterize how medical research is portrayed, including the frequency, context, accuracy, and ethical framing of research-related storylines.

Methods: A systematic content analysis was conducted of episodes from prime-time legal and medical dramas aired over the past 20 years. Episodes involving medical research were identified via streaming platforms and Internet Movie Database (IMDb), and then coded using a seven-domain framework (plot, themes, ethical dilemmas, impact on public perceptions, character portrayals, accuracy, and key developments) developed by an emergency medicine expert panel. One investigator re-reviewed a random 10% subsample to assess reliability (kappa), and results were summarized with descriptive statistics and frequency tables.

Results: A total of 170 episodes were identified from 7 legal and 20 medical dramas. *Grey's Anatomy*, *House*, and *Do No Harm* yielded the most research-focused episodes. Depictions of research were generally unfavorable (82.9%). Research protocols and informed consent were frequently misrepresented (63.5%), medical errors and cover-ups were exaggerated (28.8%), and consequences for unethical behavior were often minimized (32.9%). The reliability of data collection was substantial (κ = 0.76).

Conclusion: In this sample of prime-time legal and medical dramas, medical research was portrayed predominantly in a negative, conflict-laden light, with recurring emphasis on ethical tension, shortcuts, and patient harm. These portrayals may foster mistrust, therapeutic misconception, and unrealistic expectations, highlighting the need for more accurate, ethically grounded depictions of medical research in popular culture.

## Introduction

Television (TV) dramas shape public understanding of medicine by blending emotionally compelling narratives with repeated exposure to clinical and scientific concepts [1]. For many viewers, these programs may function as an informal curriculum on healthcare delivery, medical ethics, and biomedical innovation. When research is depicted inaccurately, the resulting messages may influence public beliefs about consent, oversight, risk, and the trustworthiness of investigators and institutions [2,3].

These portrayals matter because they reach large, diverse audiences and are often encountered long before individuals engage directly with medical care or research, potentially shaping baseline expectations about how studies are designed, reviewed, and conducted. Repeated viewing may influence how viewers perceive ethical conflict, regulatory processes, and heroic rule‑breaking, which in turn may contribute to blurring the line between acceptable clinical care and research and to therapeutic misconception or mistrust of academic‑ and industry‑sponsored studies [4,5]. Against a backdrop of broader societal concerns about misinformation and declining trust in science, understanding what messages viewers receive about research from popular media has become increasingly important [6].

Prior research has examined public health themes, professionalism, and clinical communication in television medical dramas [1,2,7], but the portrayal of medical research itself has received less direct study. Existing work suggests that shows often dramatize complex ethical issues and regulatory processes, yet systematic characterization of how research design, oversight, and participant protections are represented remains limited [2,8,9]. This study’s primary objective was to describe and classify portrayals of medical research in prime-time U.S. medical and legal dramas, focusing on evaluative tone and accuracy of depiction. Secondary objectives were to identify recurring narrative and ethical themes and to consider the potential implications of these portrayals for public understanding of research. The study evaluates media content rather than audience attitudes or behavioral outcomes and is therefore intended to describe televised narratives, not to measure their direct effects on viewers.

## Materials and methods

Study design and setting

This study used a systematic mixed-methods content analysis to examine how medical research is portrayed in U.S. prime-time medical and legal TV dramas. A content-analysis approach was selected because it allows both quantitative description of recurring research-related elements and qualitative interpretation of the ethical and narrative framing in which those elements appear [10]. The study was conducted within an academic emergency medicine research group with prior experience analyzing portrayals of medicine and professionalism in popular media.

Identification of eligible series and episodes

The sampling frame consisted of U.S. prime-time medical and legal dramas aired during the prior 20 years. Series were screened for eligibility if they were primarily drama-format programs and included one or more storylines involving medical research, such as clinical trials, experimental therapeutics, protocol-driven interventions, investigator-participant interactions, or research oversight processes. Documentary programming, reality television, non-drama formats, and series in which medical research was absent or only incidental to the narrative were excluded.

Episodes were identified through a structured search of major streaming platforms, the Internet Movie Database (IMDb), and episode-guide resources [11]. Eligible episodes were prime-time medical and legal dramas originally broadcast in the United States between 2005 and 2025 that contained a central or clearly substantive secondary storyline involving medical research. Three reviewers independently screened series and episodes using combinations of research‑related keywords (such as research, clinical trial, study, experimental, protocol, informed consent, placebo, and IRB) and review of detailed episode summaries and plot descriptions. Discrepancies in episode selection were discussed and resolved by consensus. Episodes were included when medical research constituted a central or clearly substantive secondary plotline with sufficient narrative detail to permit coding of research conduct, ethical framing, and consequences. The Preferred Reporting Items for Systematic reviews and Meta-Analyses (PRISMA)-style screening flow was not formally applied as this project was a TV-episode content analysis rather than a systematic review of published studies. However, the methods - prospective inclusion/exclusion criteria, multiple independent reviewers, and consensus resolution of disagreements - mirror core PRISMA principles around transparent study selection and screening.

Sampling and final analytic dataset

After screening, eligible episodes were assembled into a final analytic sample of 170 episodes drawn from 27 television series, including 20 medical dramas and 7 legal dramas broadcast between 2005 and 2025 (see Appendix A for a full list of included series and episode counts). *Grey's Anatomy*, *House*, and *Do No Harm* contributed the greatest number of research-focused episodes. Because the goal was to characterize recurring portrayals across a broad media landscape rather than estimate audience exposure, the study used purposive comprehensive sampling of eligible episodes rather than random selection.

Codebook development

A detailed coding manual was developed by an emergency medicine expert panel and refined iteratively before full data collection. The coding framework included seven major domains: plot, themes, ethical dilemmas, potential impact on public perceptions, character portrayals, medical or research accuracy, and key developments or outcomes. Within the themes and ethical dilemma domains, coders assessed the presence and prominence of 15 predefined thematic categories capturing consent, oversight, vulnerability, misconduct, risk-benefit framing, trust, disparities, media, and professional identity, each rated as absent, minor, or major for each episode (Appendix B). Additional abstraction elements included episode identifiers, narrative summary, research topic area, character roles, demographic and social characteristics of major characters when relevant to the storyline, perceived medical or research accuracy, responsible portrayal of research, and episode outcomes.

Overall medical and research accuracy was coded as a global ordinal rating (very inaccurate, somewhat inaccurate, mixed, mostly accurate, highly accurate, or unclear) based on how closely the depicted study design, consent process, oversight, timelines, and consequences resembled contemporary research practice, using predefined anchors in the study codebook.

The codebook was developed using both deductive and inductive approaches. Deductive categories were informed by the study objectives and prior work on television portrayals of medicine, professionalism, ethics, and public health, while inductive refinement occurred during pilot review of a subset of episodes to capture emergent themes not fully anticipated in the original framework. Operational definitions and decision rules were revised during this pilot phase to improve consistency across coders and across series with different narrative styles.

Coding procedures

All coders participated in an initial training session to review the study purpose, abstraction form, codebook definitions, and example episodes. During the pilot phase, coding rules were tested on a small group of episodes and discussed in consensus meetings to identify ambiguities, reconcile interpretive differences, and standardize application of the codebook. Once the codebook was finalized, episodes were coded using standardized abstraction forms and entered into a structured dataset for analysis.

For each episode, coders assessed both manifest content and latent narrative framing. Manifest content included observable elements such as the presence of a clinical trial, the depiction of informed consent, the involvement of an oversight body, and the occurrence of patient harm or professional sanction. Latent coding addressed broader interpretive features such as whether research was framed as exploitative, heroic, obstructive, ethically conflicted, or socially beneficial.

Primary and secondary outcomes

The primary outcome was the overall tone of the episode's portrayal of medical research, categorized as favorable, neutral, or unfavorable according to a priori coding criteria based on ethical framing, portrayal of participant protection, narrative consequences of misconduct, and the overall positioning of research within the storyline. Subjective global coding categories were operationalized in advance. For example, ‘favorable’ portrayals emphasized ethically grounded research conduct, meaningful consent, appropriate oversight, and accountability; ‘unfavorable’ portrayals emphasized ethical conflict, protocol deviation, weak accountability, and participant exploitation; and ‘responsible portrayal’ referred to depictions judged to align substantially with accepted principles of research ethics, informed consent, and oversight.

Secondary outcomes included the presence and frequency of specific themes and misrepresentations, including inaccurate depictions of research protocols, problematic informed consent processes, exaggerated medical errors or cover-ups, minimization of consequences for unethical conduct, portrayals of oversight bodies as obstructive, and narratives suggesting exploitation of vulnerable participants.

Reliability and quality assurance

To assess coding reliability, one investigator re-reviewed a random 10% subsample of episodes. Interrater agreement for the primary outcome (overall evaluative tone of the research portrayal: favorable, neutral, or unfavorable) was quantified using Cohen's kappa and was 0.76, indicating substantial agreement. In addition to formal reliability assessment, regular consensus meetings were held during the coding process to address uncertain cases, clarify definitions, and reduce drift in the application of the coding framework over time. The principal investigator also conducted periodic reviews of the collected data to identify transcription errors and reduce inter-observer variation.

Data analysis

Analyses were primarily descriptive and not powered to detect small differences between subgroups. Quantitative results were summarized using descriptive statistics and frequency tables. These analyses characterized the distribution of research-related themes, overall valence of research portrayals, and the frequency of specific inaccuracies and ethical concerns across the sampled episodes. Qualitative thematic analysis was used to identify recurrent narrative patterns in how research was framed across series and episodes. Attention was paid to themes involving informed consent, oversight, transparency, accountability for harm, and tensions between innovation and patient safety. Illustrative examples and outlier episodes were used to contextualize these themes and capture how dramas normalized rule-bending, individualized heroism, and distrust of regulatory oversight.

## Results

Sample characteristics

The final sample comprised 170 episodes from 27 series broadcast between 2005 and 2025. Research-related storylines were coded as central plots in 100 episodes (58.8%) and as substantive secondary plots in 70 (41.2%). The most frequently represented series were *Grey’s Anatomy*, *House*, and *Do No Harm*, which together accounted for 77 episodes (45.3%). The most common research modality was clinical investigation (formal clinical trials and one-off experimental interventions), followed by psychiatric and behavioral studies, public health investigations related to outbreaks, and less frequent genetics or precision-medicine storylines.

Overall evaluative tone of the research portrayal

Across 170 episodes, the overall evaluative tone of the medical research portrayal was coded as unfavorable in 141 episodes (82.9%), neutral in 9 (5.3%), and favorable in 20 (11.8%). Interrater agreement for this primary outcome was substantial, with Cohen’s kappa of 0.76 on a 10% random subsample.

In episodes coded as unfavorable, research frequently involved conflict between investigators’ professional or institutional aims and patient welfare, and decisions to bend or break protocols under time pressure. Oversight bodies were often present but depicted as obstructive or out of touch, and many storylines included harms, concealment of adverse events, or delayed responses to misconduct.

In episodes coded as favorable, research was more often framed as a structured extension of patient care and scientific inquiry. Investigators typically discussed uncertainty and risks in more detail, consent processes appeared more deliberate, and oversight structures were portrayed as participating in protocol review and participant protection. These episodes more often ended with clear benefits or constructive lessons from complications.

Accuracy, misrepresentation, and oversight

Medical and research accuracy was rated as high or mostly accurate in 19 episodes (11.2%), mixed in 24 (14.1%), and somewhat or very inaccurate in 127 (74.7%). Research was portrayed responsibly in 15 episodes (8.8%), while 155 (91.2%) depicted research in a predominantly irresponsible or misleading manner.

Specific misrepresentations were common. Inaccurate or problematic depictions of research protocols and informed consent occurred in 108 episodes (63.5%). Exaggerated medical errors or cover-ups appeared in 49 episodes (28.8%), and consequences for unethical behavior were minimized or delayed in 56 (32.9%). Oversight bodies such as IRBs, hospital committees, or regulators appeared in 67 episodes (39.4%), most often framed as obstructive or political rather than protective. Consent was frequently brief, incomplete, or obtained under clear distress or pressure, and protocol deviations were undertaken with limited reference to safeguards.

Thematic findings

Consent, Vulnerability, and Protections

Episodes clustered around recurring themes, including informed consent, oversight and regulation, participant protections and vulnerability, risk-benefit framing, and the “hidden curriculum” of heroism and rule-breaking (Table 1).

**Table 1:** Major themes in portrayals of medical research in TV dramas

Theme	Brief description
Ethics as conflicted	Research is framed as ethically unstable, with investigators repeatedly torn between professional ambition, institutional pressure, and duties to individual patients.
Ambition versus patient care	Storylines pit clinician-investigators’ drive for innovation or career advancement against immediate obligations to patient welfare at the bedside.
Normalized rule-bending	Rule-breaking and protocol shortcuts are depicted as necessary, even admirable, moves to save lives or achieve dramatic breakthroughs.
Compromised consent	Informed consent appears rushed, manipulative, or easily bypassed when inconvenient, often under conditions of urgency, desperation, or unequal power.
Weak consequences for misconduct	Unethical or harmful research behaviors rarely lead to timely, meaningful professional, institutional, or legal consequences.
Dramatic harms and cover-ups	The narratives highlight dramatic errors, cover-ups, and retaliation against whistleblowers far more than routine, safe, protocol-driven practice.
Oversight as obstruction	Oversight bodies such as IRBs, hospital committees, or regulators are portrayed as obstructive, political, or out of touch rather than as safeguards.
Maverick heroism over teamwork	Stories emphasize maverick individual heroism and lone-wolf decision-making instead of collaborative, team-based research and shared accountability.
Exploited vulnerability	Research participation is closely associated with exploiting vulnerable or desperate patients, including those with few alternatives or limited resources.

Themes related to informed consent and voluntariness were present in 34 episodes (20.0%), with major consent issues coded in 19 (11.2%). Participant vulnerability and protections were salient in 44 episodes (25.9%), often involving socioeconomically disadvantaged, critically ill, or otherwise marginalized patients. In these episodes, consent decisions were frequently depicted under conditions of urgency or marked power imbalance.

Exploitation and injustice were major themes in 33 episodes (19.4%), often in the context of patients with few alternatives or groups with a history of marginalization. Health disparities and access-related themes appeared in 23 episodes (13.5%), with research participation sometimes portrayed as a primary route to advanced care.

Oversight, Misconduct, and Rule-Breaking

Oversight and regulation themes appeared in 59 episodes (34.7%). In 53 of 59 (89.8%), oversight bodies were framed primarily as obstructive or politicized; in 6 of 59 (10.2%), they functioned mainly as safeguards. Protocol adherence and rule-breaking themes were present in 67 episodes (39.4%). Within this subset, rule-breaking by a clinician-investigator was commonly depicted as central to the storyline, with accountability occurring late or only partially. Explicit research misconduct or cover-ups were major themes in 44 episodes (25.9%), including concealment of adverse events, suppression of negative data, and intimidation of whistleblowers.

Risk-Benefit Framing, Trust, and Public Impact

Innovation versus patient safety was a major theme in 48 episodes (28.2%), frequently in high-risk interventions undertaken under time pressure or in last-resort scenarios. Risk-benefit framing was skewed toward overstating benefit and/or understating risk in 29 episodes (17.1%), whereas a more balanced discussion of uncertainty was observed in 9 episodes (5.3%).

Trust in research and institutions was an explicit or implicit positive theme in 7 episodes (4.1%), whereas 126 episodes (74.1%) explicitly or implicitly promoted mistrust of investigators, hospitals, or the pharmaceutical industry. Public health or population-level impact of research appeared in 19 episodes (11.2%), particularly in pandemic or outbreak storylines, which often depicted rapid development and deployment of interventions.

Communication, Identity, and Hidden Curriculum

Media, communication, and public understanding of research were addressed in 13 episodes (7.6%), including depictions of news coverage, hospital public relations, and explanatory conversations with patients or families. Professional identity, training, and moral distress among clinicians or trainees were salient in 22 episodes (12.9%), often highlighting tension between clinician and investigator roles.

Themes of heroism, collaboration, and a hidden curriculum of research appeared in 30 episodes (17.6%). These narratives frequently emphasized individual heroism and rule-breaking over team-based, protocol-driven practice.

Character and Storyline Patterns

Investigators were most often depicted as attending physicians, subspecialists, or principal investigators in 111 episodes (65.3%), while trainees (fellows, residents, or students) had prominent roles in 61 (35.9%). Patients were frequently portrayed as vulnerable subjects in 60 episodes (35.3%). Research storylines concluded with clear benefits in 58 episodes (34.1%), clear harm in 27 (15.9%), mixed or ambiguous outcomes in 30 (17.6%), and unresolved outcomes in 55 (32.3%).

Institutional responses to adverse events or misconduct were observed in 44 episodes (25.9%), with no meaningful consequence shown in 13 episodes (7.6%), internal review or informal sanction in 11 (6.5%), and formal legal or regulatory consequences in 20 (11.8%).

Qualitative Synthesis of Narrative Framing

Qualitative thematic analysis showed that research was most often framed as conflict-laden and ethically unstable, with repeated tension between innovation and patient safety, oversight and clinical urgency, and individual heroism and institutional rules. Illustrative episodes highlighted consent challenges, oversight portrayed as obstruction, and frequent association of research participation with risk and exploitation.

A smaller subset of episodes provided more balanced or positive portrayals, emphasizing careful consent processes, patient advocacy, collaborative decision-making, and transparent oversight. These outlier narratives indicate that some dramas depicted research as ethically grounded and procedurally robust while maintaining dramatic tension.

## Discussion

In this mixed-methods content analysis of 170 research-focused episodes from U.S. prime-time medical and legal dramas, the primary objective was to characterize how medical research was portrayed. The findings showed that portrayals were predominantly unfavorable and frequently emphasized ethical conflict, protocol deviation, weak accountability, and patient vulnerability. Rather than depicting research as a structured, collaborative, and highly regulated enterprise, many narratives located it in a morally unstable space characterized by secrecy, rule-bending, and high-stakes heroism. These patterns suggest that television dramas often foreground the most conflict-laden aspects of research practice while downplaying routine safeguards [8,12].

Several recurring patterns are especially relevant to the study’s secondary objective of identifying narrative and ethical themes with possible implications for public understanding of research. In many episodes, informed consent was often depicted as superficial, rushed, manipulative, or easily bypassed, with participant protections overshadowed by urgency, desperation, or pronounced power imbalances. Oversight bodies such as IRBs, hospital committees, and regulators were more likely to appear as obstructive or politicized barriers than as mechanisms of protection. Rule-breaking by well-intentioned clinicians was commonly framed as morally justified, reinforcing the impression that ethical shortcuts are necessary when lives are at stake.

Taken together, these patterns convey several lessons for educators, institutions, and science communicators. First, rule-bending is frequently normalized when “good doctors” are shown violating protocols to help patients, while exaggerated harms and limited consequences obscure real accountability structures [7,8]. Second, dramas frame research as inherently conflict-laden, highlighting risk, liability, and bureaucracy more than participant protection and routine safe practice [9]. Third, because medical dramas serve as a lay source of information, there is a need for proactive communication to address misconceptions and for collaboration among writers, ethicists, and investigators to depict safeguards, team-based decision-making, and iterative ethical review without sacrificing dramatic tension.

Because this study evaluates media content rather than audience attitudes, comprehension, or behavior, these interpretations should be understood as plausible implications of the observed portrayals rather than demonstrated effects on viewers. This narrative structure may matter because television functions as an informal source of health and science education for many viewers. Repeated exposure to storylines in which research participation is associated with exploitation, secrecy, or institutional self-interest may contribute to mistrust of investigators, hospitals, and industry, and may blur distinctions between individualized clinical care and protocol-driven research in ways that could foster therapeutic misconception [13,14]. The relative rarity of trust-oriented portrayals, compared with the frequency of mistrust-oriented narratives, suggests a pattern that may tilt audience impressions toward suspicion rather than nuance, although that possibility was not directly measured in this study.

The findings also have implications for medical education, science communication, and public engagement [7,8,15]. Research ethics, participant protections, and the role of oversight can be challenging to teach abstractly; these televised narratives may provide vivid case material for structured discussion with students, trainees, and community audiences. At the same time, educators and institutions may need to address media-derived misconceptions more explicitly in patient education, outreach, and research recruitment materials [16,17]. Collaborations among showrunners, ethicists, and clinical investigators could preserve narrative tension while more faithfully depicting consent, accountability, and team-based decision-making [1,8,12].

Notably, a smaller subset of episodes offered more balanced portrayals. These episodes showed more deliberate consent processes, meaningful oversight, transparent communication, and research that generated either a credible benefit or a realistic presentation of uncertainty. These outlier narratives suggest that it is possible to maintain substantial dramatic tension while depicting research as ethically grounded and procedurally robust. Highlighting and building on such examples may offer a constructive pathway for collaborations between clinicians, ethicists, and media creators.

Limitations

This study has several limitations. First, as a content analysis of scripted entertainment media, it can characterize portrayals of research but cannot determine whether those portrayals directly influence viewer beliefs, attitudes, or behaviors. The study, therefore, identifies plausible pathways by which television might shape public understanding, but it does not measure audience reception or causal effects [1].

Second, although the sampling strategy was broad and purposive, it was limited to U.S. prime-time medical and legal dramas broadcast between 2005 and 2025 and to episodes in which medical research constituted a central or clearly substantive secondary storyline. These findings may not generalize to non-U.S. programming, streaming-only genres outside the sampling frame, documentaries, reality television, or dramas in which research is present only peripherally. In addition, the sampling strategy was purposive rather than probabilistic, targeting series and episodes with substantive research storylines. This approach is appropriate for identifying and comparing narrative patterns but may over-represent programs that foreground research and under-represent shows in which research appears more peripherally or is depicted in a less dramatic fashion.

Third, several coding decisions necessarily involved interpretive judgment, particularly for latent constructs such as overall tone, responsible portrayal, mistrust, or the ethical framing of oversight. Although coder training, pilot refinement, consensus meetings, and a kappa of 0.76 support acceptable reliability, some degree of subjective interpretation is inherent in this methodology.

Fourth, the study evaluated portrayals at the episode level rather than measuring audience reach, cumulative exposure, or the cultural influence of specific series. As a result, episodes from highly watched programs and episodes from less prominent series contributed equally to the analytic sample, which is appropriate for describing narrative patterns but not for estimating real-world exposure to those messages.

Finally, the abstraction framework was designed to capture a wide range of research-related themes across heterogeneous series and narrative styles. That breadth improves descriptive coverage but may compress important distinctions between types of research, such as formal clinical trials, ad hoc experimental interventions, translational research, and public health investigations. In addition, although coding disagreements were resolved by consensus, investigators’ clinical, research, and educational backgrounds may still have influenced latent interpretation of narrative tone, ethical framing, and realism. Future work could extend these findings by pairing content analysis with audience studies, weighting episodes by viewership, and comparing portrayals across genres, eras, and platforms.

## Conclusions

Medical research in U.S. prime-time medical and legal dramas was portrayed predominantly as conflict-laden, ethically unstable, and only weakly constrained by oversight, with frequent misrepresentation of informed consent, protocol adherence, and accountability. Across this sample, research was far more likely to be framed as a source of mistrust, urgency, and institutional conflict than as a structured, collaborative, and highly regulated process. These portrayals may contribute to therapeutic misconception, normalize ethical shortcuts, and erode confidence in participant protections and research oversight, although audience attitudes and behaviors were not directly measured in this study.

A greater collaboration among media creators, ethicists, and clinical investigators may help support portrayals that remain dramatically compelling while more accurately representing the consent process, the role of oversight bodies, and the team-based nature of modern biomedical research. The presence of a smaller subset of balanced, accurate episodes in this sample demonstrates that responsible depictions of research ethics are compatible with engaging storytelling and suggests a feasible pathway for improving portrayals without sacrificing dramatic tension.

## Appendices

Appendix A

**Table 2:** Television series included in the sampling frame

Series title	Genre	Network/platform	Years sampled	Episodes screened	Episodes included
Grey's Anatomy	Medical	ABC	2005-2025	448	45
The Good Doctor	Medical	ABC	2017-2025	126	12
The Knick	Medical	Cinemax	2014-2015	20	4
Getting On	Medical	HBO	2013-2015	18	5
Heartbeat	Medical	NBC	2016	10	3
Pure Genius	Medical	CBS	2016-2017	13	8
Do No Harm	Medical	NBC	2013	13	13
3 lbs	Medical	CBS/Amazon	2006	8	1
Virgin River	Medical	Netflix/Faith & Family	2019-2025	64	1
ER	Medical	NBC	2005-2009	108	4
New Amsterdam	Medical	NBC	2018-2023	89	1
Chicago Med	Medical	NBC	2015-2025	198	10
The Resident	Medical	Fox	2018-2023	107	6
Code Black	Medical	CBS	2015-2018	47	5
9-1-1	Medical	Fox/ABC	2018-2025	142	1
The Night Shift	Medical	NBC	2014-2017	45	1
House	Medical	Fox	2005-2025	155	19
Saving Hope	Medical	NBC	2012	12	8
Royal Pains	Medical	USA Network	2009-2016	104	3
Scrubs	Medical	ABC	2005-2010	114	4
Suits	Legal	USA Network	2011-2019	134	1
Goliath	Legal	Amazon	2016-2021	32	1
Bull	Legal	CBS	2016-2022	125	3
Law & Order	Legal	NBC	2005-2025	174	2
Law & Order: SVU	Legal	NBC	2005-2025	434	4
Boston Legal	Legal	ABC	2005-2008	84	4
The Firm	Legal	NBC	2012	22	1

Appendix B

**Table 3:** Primary themes for coding medical research portrayals

Primary theme	Operational description
Informed consent and voluntariness	Whether consent is obtained, the quality of consent (for example, thorough versus rushed), and issues of coercion or undue influence.
Oversight and regulation	IRBs, ethics committees, hospital boards, regulators, and how these bodies are framed, such as safeguards versus obstacles.
Participant protections and vulnerability	Privacy protections, safety monitoring, vulnerable populations, and respect for withdrawal or refusal.
Conflicts of interest and power dynamics	Financial, professional, or personal conflicts, and imbalances of power between investigators, institutions, and participants.
Protocol adherence and rule-breaking	Shortcuts, off-protocol actions, unauthorized changes, and whether rule-breaking is normalized or rewarded.
Research misconduct and cover-ups	Fraud, data falsification, suppression of adverse events, concealment, intimidation of whistleblowers, and explicit cover-up narratives.
Innovation versus patient safety	Tension between urgency or innovation and caution or safety, including “nothing left to lose” narratives and high-risk experimentation.
Risk, benefit, and outcomes of research	How risks are described, benefits promised or realized, severity of harms, and the balance between individual and societal benefit.
Trust and mistrust in research and institutions	Explicit or implicit messages that promote or undermine trust in investigators, hospitals, academia, industry, or regulators.
Exploitation and injustice	Exploitation of desperate, marginalized, or socioeconomically vulnerable participants, and unfair treatment or manipulation.
